# Removing mental health barriers to education: A community project

**DOI:** 10.4102/sajpsychiatry.v24i0.1293

**Published:** 2018-09-27

**Authors:** Renata Schoeman, Jena Enright, Anelet James, Cornelia Vermeulen, Nic de Beer

**Affiliations:** 1 University of Stellenbosch Business School, Stellenbosch University, South Africa; 2 Goldilocks and The Bear Foundation, South Africa

## Abstract

**Introduction:**

Attention-deficit hyperactivity disorder (ADHD) is the most common psychiatric disorder in children – affecting 2% to 16% of the school-age population (National Resource Centre on AD/HD, 2013). However, in South Africa, data on prevalence rates, access to care and treatment for ADHD are limited and research is lacking. Many children- especially those in underprivileged communities- suffering from ADHD remain undiagnosed, or if diagnosed, do not receive optimal treatment. The Goldilocks and The Bear Foundation provides screening for ADHD and other mental health disorders to learners at school level. We will be presenting the statistics compiled for the first year of operation (July 2017 – June 2018).

**Methods:**

In the schools visited, children are referred to the Foundation by the School Based Support. Collateral information is obtained from educators and parents, and parental consent secured before any child is evaluated. Trained nurses do the basic physical screening and behavioural observations for each child. All information is collated and controlled by a trained psychiatrist or psychologist, who determine the possible diagnosis and refer accordingly (either to the community clinic, school doctor, optometrists, audiologists, educational psychologist or occupational psychologist). A detailed database was built capturing all the aforementioned information.

**Results:**

We have visited 18 schools (N = 12 447), of which 13 schools participated (N = 8780). A total of 543 children (6.2%) from the school population were screened. The ratio of boys to girls was 2:1, with an age range of 5–14 years. Of the children included in the current analysis (to be updated at the end of the study period), 2.7% were diagnosed with ADHD and 0.67% with depression and/or anxiety. Further detail will be provided with regard to risk factors and comorbid conditions and problem areas.

**Conclusion:**

Although mental health clinics exist in the public sector, children with ADHD often never reach this point of diagnosis and treatment because of a lack of awareness and knowledge in their communities. Improved outcomes are possible to achieve if patients suffering from ADHD are diagnosed as such and receive multi-modal intervention – which would include psychopharmacological interventions, behavioural interventions and support.

